# Analysis of Important Gene Ontology Terms and Biological Pathways Related to Pancreatic Cancer

**DOI:** 10.1155/2016/7861274

**Published:** 2016-11-09

**Authors:** Hang Yin, ShaoPeng Wang, Yu-Hang Zhang, Yu-Dong Cai, Hailin Liu

**Affiliations:** ^1^Department of Gastroenterology, Ninth People's Hospital, School of Medicine, Shanghai Jiao Tong University, Shanghai 200011, China; ^2^School of Life Sciences, Shanghai University, Shanghai 200444, China; ^3^Institute of Health Sciences, Shanghai Institutes for Biological Sciences, Chinese Academy of Sciences, Shanghai 200031, China

## Abstract

Pancreatic cancer is a serious disease that results in more than thirty thousand deaths around the world per year. To design effective treatments, many investigators have devoted themselves to the study of biological processes and mechanisms underlying this disease. However, it is far from complete. In this study, we tried to extract important gene ontology (GO) terms and KEGG pathways for pancreatic cancer by adopting some existing computational methods. Genes that have been validated to be related to pancreatic cancer and have not been validated were represented by features derived from GO terms and KEGG pathways using the enrichment theory. A popular feature selection method, minimum redundancy maximum relevance, was employed to analyze these features and extract important GO terms and KEGG pathways. An extensive analysis of the obtained GO terms and KEGG pathways was provided to confirm the correlations between them and pancreatic cancer.

## 1. Introduction

Pancreatic cancer has been widely reported as a malignant tumor subtype involving one of the most significant tissue organs that contribute to both digestive system and endocrine system, the pancreas. Based on clinical symptoms and genetic characteristics, pancreatic cancer can be clustered into various subtypes [1]. Among such subtypes, pancreatic ductal adenocarcinoma (PDAC) accounts for more than 90% of all the cases. With a specific low survival rate (18% for one-year survival rate and 5% for five-year survival rate), pancreatic cancer results in more than thirty thousand deaths around the world and has been regarded as one of the top killers for human beings [1, 2].

Although pancreatic cancer has been included in the list of top killers for human beings, the biological processes and mechanisms that contribute to the initiation and progression of pancreatic cancer have not been fully revealed. Based on recent publications, the underlying mechanisms of pancreatic cancer have been partially uncovered mainly by experimental trials [3, 4]. The traditional experimental trials that contribute to revealing of pancreatic cancer associated genes and pathways can be divided into two levels: the nucleotide level (DNA/RNA) and the protein level. At the nucleotide level, polymerase chain reaction (PCR), high-throughput sequencing, and gene chips (either genomic chips or expression profile chips) contribute to the identification of the genomic and transcriptional background for pancreatic cancer initiation and progression [5]. Taking gene chip as an example, such experimental tool reveals the detailed genetic and expression profile characteristics of tumor cells and has been reported to contribute to the identification of various pancreatic cancer associated biological processes, including DPC4 tumor-suppressor pathway and the famous MAPK signaling pathway which we will analyze below [6–8]. As for the protein level, western blot turns out to be the most commonly used biochemical method to identify the expression and activation status of a known protein in certain in vivo or in vitro environment. Further, relying on in vitro gene expression (RNA) interference technologies, the characteristic alteration of the expression and function of a series of proteins that have been identified on such two levels as we have mentioned above can be validated and such group of proteins can be further concluded into various biological processes and pathways [9, 10]. Based on the experimental technologies we have mentioned above, various principal regulatory pathways have been identified and confirmed to contribute to the initiation and progression of pancreatic cancer.

Based on existing publications, various principle regulatory pathways and biological processes that contribute to the initiation and progression of pancreatic cancer have been identified. Such signaling pathways and biological processes contribute to three main aspects of the biological processes of pancreatic cancer: transmembrane signal transduction, intracellular metabolic transduction, and the intranuclear proliferative regulation [3, 11, 12]. Different signaling pathways have been identified to contribute to different biological processes of pancreatic cancer during tumorigenesis. According to recent literatures, ErbB signaling pathway and TGF-beta signaling pathway participate in the transmembrane signal transduction of pancreatic cancer [13, 14]. Such transmembrane signal transduction pathways have been further validated to transfer the signals to intracellular pathways (such as p53 signaling pathway, MAPK signaling pathway, PI3K-Akt signaling pathway, and VEGF signaling pathway) [13, 15–17]. Intracellular signaling pathways have been identified to contribute to the abnormal proliferation of pancreatic cells and further initiate the tumorigenesis. Taking MAPK signaling pathway as an example, as the downstream region of Ras signaling pathway, MAPK signaling pathway contributes to the phosphorylation of two crucial families of proteins, ERK and JNK, and further regulates proliferative signaling transportation into the nucleus [8]. Although various functional pathways have been revealed to contribute to the abnormal proliferation during the tumorigenesis of pancreatic cancer, the core trigger for the initiation of pancreatic cancer turns out to be the abnormal intranuclear proliferative regulation [18]. It has been identified that two main biological processes contribute to the abnormal proliferation of pancreatic cells during tumorigenesis: the inhibition of cell apoptosis and the excessive activation of proliferation [19]. All of such regulatory signaling pathways have been reported to be abnormal in pancreatic cancer and further contribute to the tumorigenesis. However, according to such signaling pathways, we still cannot explain all the pathological phenotypes of pancreatic cancer, implying that there are still core regulatory pathways remaining to be uncovered.

The study on the underlying mechanism of pancreatic cancer has lasted for decades [20]. However, based on experimental methods, only limited genes and pathways are proved to contribute to pancreatic cancer. The experimental methods that contribute to the identification and confirmation of pancreatic cancer associated pathways are quite expensive and time-consuming. Recently, with the development of computational biology and bioinformatics, various computational methods have been presented to predict cancer, including pancreatic cancer associated genes [21]. However, up to now, few computational methods have been present to describe the detailed functional pathways and biological processes of pancreatic cancer. In computational biology, KEGG pathways and gene ontology (GO) terms are widely used to describe the detailed and specific biological processes in human cells. KEGG (Kyoto Encyclopedia of Genes and Genomes) has been widely regarded as an integrated database resource for gene and protein annotation [22]. Based on KEGG database, we can obtain the KEGG pathway maps which reflect the functional pathway based network in living cells [22]. On the other hand, GO is a bioinformatics initiative to unify the presentation of gene and gene product attributes across all species [23]. Therefore, KEGG pathways and GO terms can provide a more accurate and clearer panorama for the underlying biological processes of pancreatic cancer.

In this study, we applied a popular feature selection method, minimum redundancy maximum relevance (mRMR) [24], to extract a group of pancreatic cancer associated KEGG pathways and GO terms, filling the gaps of current study in pancreatic cancer. First, genes that have been validated to be related to pancreatic cancer were deemed positive samples, while other genes were deemed negative samples. Second, the enrichment theory of GO term and KEGG pathway was adopted to encode each gene. Third, all GO terms and pathways were analyzed by mRMR method and some of the important ones were extracted. Finally, the extracted GO terms and KEGG pathways were extensively discussed to confirm their relationships to pancreatic cancer.

## 2. Materials and Methods

### 2.1. Materials

The validated genes related to pancreatic cancer were retrieved from the KEGG pathway, which is a main database in KEGG database [25]. 65 validated genes were extracted from the pathway hsa05212 (http://www.genome.jp/kegg-bin/show_pathway?map=hsa05212&show_description=show, accessed in December 2014). These genes were termed as positive samples, comprising the gene set *S*_*p*_, and are listed in Supplementary Material I available online at http://dx.doi.org/10.1155/2016/7861274. To extract the GO terms and KEGG pathways that are specific to pancreatic cancer, it is necessary to employ some genes that are not related to pancreatic cancer. Since we used the enrichment scores of GO terms and KEGG pathways to indicate the associations between genes and GO terms (KEGG pathways), genes without these scores were not considered in this study. Up to now, there are 18,600 genes whose GO and KEGG enrichment scores can be calculated. Beside the 65 genes related to pancreatic cancer, each of the remaining 18,535 genes can be deemed a negative sample because the probability of it being related to pancreatic cancer is not very high. These 18,535 genes comprised the gene set *S*_*n*_. The whole gene set *S* was constructed by combining the genes in *S*_*p*_ and *S*_*n*_; that is, *S* = *S*_*p*_ ∪ *S*_*n*_.

### 2.2. Feature Construction

To extract important GO terms and KEGG pathways that are related to pancreatic cancer, it is necessary to encode each gene in *S* based on all GO terms and KEGG pathways. Here, we used the enrichment theory of GO term and KEGG pathway to encode each gene, which can indicate the relationships between genes and GO terms (KEGG pathways). Then, the difference between positive and negative samples can be distinguished by the key features produced by a feature selection method, which would be described in Section 2.3. The encoding procedure is shown as follows.

*GO Enrichment Score*. The GO enrichment score was utilized to represent the quantitative correlation between each GO term and involved genes. For a given GO term GO_*j*_ and a gene *g*, let *G*_1_ be a gene set consisting of genes annotated to GO_*j*_ and *G*_2_ be another gene set consisting of neighbor genes of *g* in the protein-protein interaction network reported in STRING (http://string-db.org/) [26], a well-known public database providing known and predicted protein-protein interactions. The GO enrichment score between GO_*j*_ and *g* is defined as the −log_10_ of the hypergeometric test *P* value [27–30] of *G*_1_ and *G*_2_, which can be calculated by

(1) $$\begin{array}{c} ES_{GO}\left(\;g,GO_{j}\;\right)=-\log_{10}\left(\;\overset{n}{\underset{k=m}{\sum }}\frac{\left(\begin{array}{c} M\\ k \end{array}\right)\left(\begin{array}{c} N-M\\ n-k \end{array}\right)}{\left(\begin{array}{c} N\\ n \end{array}\right)}\;\right), \end{array}$$

where *N* is the number of genes in human, *M* is the number of genes in *G*_1_, *n* is the number of genes in *G*_2_, and *m* is the number of common genes of *G*_1_ and *G*_2_. A large enrichment score between GO_*j*_ and *g* indicates close relationship between them. In this study, we considered 12,511 GO terms, inducing 12,511 GO enrichment scores for each gene, which can be obtained by an in-house program using R function phyper. The R code is “score ← −log10(phyper(numWdrawn − 1, numW, numB, numDrawn, lower.tail=FALSE)),” where numW, numB, and numDrawn are the number of genes annotated to GO_*j*_, the number of genes not annotated to GO_*j*_, and the number of neighbors of gene *g* and numWdrawn is the number of neighbors of gene *g* that are also annotated to GO_*j*_.

*KEGG Enrichment Score*. Similar to that of the GO terms, the relationship between KEGG pathways and genes in *S* can be represented by the KEGG enrichment scores. For a given KEGG pathway *K*_*j*_ and a gene *g*, let *G*_1_ be a gene set consisting of genes in *K*_*j*_ and *G*_2_ is same as *G*_2_ in the above paragraph. The KEGG enrichment score between *K*_*j*_ and *g* is also defined to be the −log_10_ of the hypergeometric test *P* value [29–31] of *G*_1_ and *G*_2_, which can be computed by

(2) $$\begin{array}{c} ES_{KEGG}\left(\;g,K_{j}\;\right)=-\log_{10}\left(\;\overset{n}{\underset{k=m}{\sum }}\frac{\left(\begin{array}{c} M\\ k \end{array}\right)\left(\begin{array}{c} N-M\\ n-k \end{array}\right)}{\left(\begin{array}{c} N\\ n \end{array}\right)}\;\right), \end{array}$$

where the definitions of *N*, *M*, *n*, and *m* are same as those in (1). Also, a high score between a KEGG pathway *K*_*j*_ and a gene *g* indicates they have strong associations. Here, we considered 239 KEGG pathways, resulting in 239 KEGG enrichment scores for each gene, which can also be obtained by an in-house program using R function phyper.

As mentioned above, each gene *g* was represented by 12,511 features derived from GO terms and 239 features derived from KEGG pathways, which can be formulated as a vector

(3) fg=ESGOg,GO1,…,ESGOg,GO12511,ESKEGGg,K1,…,ESKEGGg,K239T.

### 2.3. Feature Selection Method

As described in Section 2.2, each gene was represented by 12,750 features derived from the GO terms and KEGG pathways. Considering the unequal roles of these features on pancreatic cancer, that is, some features playing more important roles than others, it is necessary to employ some advanced tools to analyze them, thereby extracting key features that are strongly associated with pancreatic cancer. Here, a reliable and widely used feature selection method, namely, mRMR method [24], was adopted to analyze all investigated 12,750 features. The mRMR method was proposed by Peng et al. [24] and was deemed to be a useful tool to analyze the feature space of complicated problems. Up to now, it has been widely applied to analyze various complicated biological systems or problems [32–45].

The mRMR method has two excellent criteria: Max-Relevance and Min-Redundancy. The criterion of Max-Relevance measures the importance of features based on their correlation to targets, while the criterion of Min-Redundancy gives a guarantee that the selected features have minimum redundancies. It is clear that the former criterion can be used to extract important features for a classification problem, while if one tries to construct an optimal feature subspace, two of them should be used. Because the purpose of this study is to extract key features that are closely related to the pancreatic cancer but not to construct an optimal feature subspace, we only used the Max-Relevance in this study. For each feature, let *f* be a variable representing values of all samples under the feature and *c* be the target variable. The mutual information (MI) value of each feature can be computed by

(4) $$\begin{array}{c} I\left(\;c,f\;\right)=\iint p\left(\;c,f\;\right)\log\frac{p\left(c,f\right)}{p\left(c\right)p\left(f\right)}dc\;df, \end{array}$$

where *p*(*c*) and *p*(*f*) are the marginal probabilities of *c* and *f*; *p*(*c*, *f*) is the joint probabilistic distribution of *c* and *f*. In fact, MI measures the mutual dependence between two variables. Furthermore, it has wide applications because it can deal with random variables that are not real-valued. Thus, mRMR method adopted MI to measure the relevance of each feature. According to the MI values of all features, a feature list, namely, MaxRel feature list, can be built. The MaxRel feature list was formulated as

(5) $$\begin{array}{c} F_{M}=\left[\;f_{1}^{M},f_{2}^{M},\ldots ,f_{N}^{M}\;\right], \end{array}$$

where *N* represented the total number of features. Clearly, features with high ranks in this list are more likely to be related to pancreatic cancer. Extensive investigation of the corresponding GO terms and KEGG pathways may give new insights for the study of pancreatic cancer.

## 3. Results and Discussion

**Figure 1:**
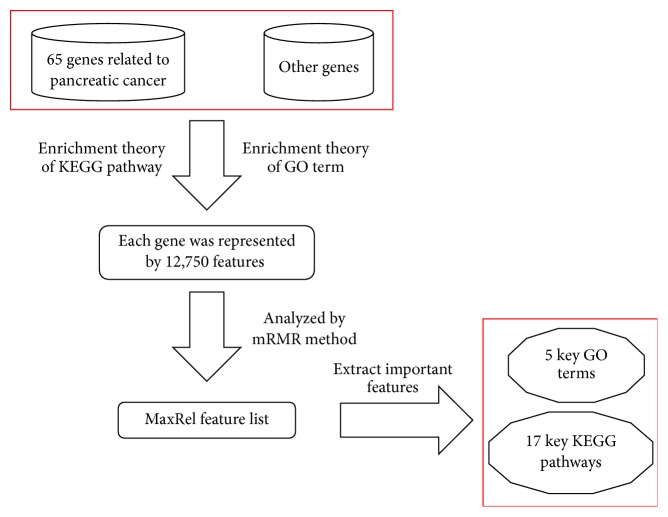
The procedures for extracting important KEGG pathways and GO terms of pancreatic cancer.

The purpose of this study is to extract important KEGG pathways and GO terms of pancreatic cancer using some computational methods. The detailed procedures are illustrated in Figure 1.

### 3.1. Results

As described in Section 2.2, each gene in *S* was represented by 12,750 features derived from the GO terms and KEGG pathways. These features were analyzed by mRMR method described in Section 2.3 by calculating their relevance to targets measured by their MI values. According to the MI value of each feature, the MaxRel feature list was constructed, which is provided in Supplementary Material II.

It is known that not all GO terms and KEGG pathways have strong associations with pancreatic cancer. The rank of a corresponding feature in the MaxRel feature list for a GO term or a KEGG pathway indicates its importance for pancreatic cancer. Thus, we can select the GO terms and KEGG pathways whose features received high ranks in the MaxRel feature list to investigate their importance. Here, we chose the 22 features receiving MI values no less than 0.01 for further analysis, resulting in 22 GO terms or KEGG pathways. Their detailed information is listed in Tables 1 and 2. It can be observed from these two tables that there are 17 important KEGG pathways (listed in Table 1) and five key GO terms (listed in Table 2). In the following section, detailed discussion on these GO terms and KEGG pathways would be given.

### 3.2. Analysis of Key KEGG Pathways and GO Terms

As shown in Tables 1 and 2, 17 KEGG pathways and five GO terms were extracted, which are deemed to be highly related to pancreatic cancer. According to recent published literature, all of these KEGG pathways and GO terms identified in this study have been confirmed to participate in pancreatic cancer associated biological processes.

#### 3.2.1. KEGG Pathways Associated with Pancreatic Cancer

17 KEGG pathways were extracted in this study, which are deemed to be associated with the initiation and progression of pancreatic cancer.

*(1) Pathways Describe Various Tumor Subtypes*. Among the 17 KEGG pathways, 10 KEGG pathways describe the whole metabolic regulatory network of a specific cancer subtype. KEGG pathway* hsa05200* describes the kernel regulatory factors that contribute to the initiation and progression of pan-cancer. Various pathways (e.g., Wnt signaling pathway, cAMP signaling pathway, and VEGF signaling pathway) in such network (hsa05200) and functional genes (e.g.,* PKA*,* Rho*, and* VEGF*) have been identified in pancreatic cancer [46–48]. Taking gene* PKA* and its corresponding signaling pathway, the cAMP signaling pathway, as an example, cyclic AMP associated pathway and* PKA* have been identified and confirmed to contribute to the migration and invasion of pancreatic cancer, validating our prediction [48].

Apart from the KEGG pathways describing the pan-cancer, various KEGG pathways have also been predicted to describe the detailed subtypes of cancer. Among them,* hsa05223* which describes the regulatory network and pathways of non-small-cell lung cancer has been predicted to be related to the specific biological processes of pancreatic cancer. Such KEGG pathway contains various tumor associated factors and pathways (such as* KRAS*,* TP53*, and functional pathways that they participate in). It has been proved that* KRAS* and* TP53* as we have mentioned above have both been reported and confirmed to contribute to the initiation and progression of pancreatic cancer [49, 50]. Considering factors like* KRAS* and* TP53* which have been identified in both pancreatic cancer associated pathways and non-small-cell lung cancer associated pathways, such two regulatory networks (pancreatic cancer and non-small-cell lung cancer associated pathways) may definitely interact with each other, and our predicted KEGG term hsa05223 may actually participate in pancreatic associated pathways validating the accuracy and efficacy of our prediction. Apart from non-small-cell lung cancer, another four subtypes of cancer (prostate cancer, endometrial cancer, renal cell carcinoma, and colorectal cancer) associated biological processes have also been predicted to be associated with pancreatic cancer. There are various core regulatory factors and pathways in prostate cancer associated pathways (*hsa05215*). Steroid hormone biosynthesis has been reported to contribute to the metastasis of prostate cancer and interacts with the specific oncogene of pancreatic cancer* AR*, implying its core role for the prostate associated pathways that we have predicted [51, 52]. According to recent publications, such core pathway of the steroid hormone biosynthesis may also contribute to pancreatic cancer, revealing the underlying relationships between prostate cancer associated pathways (as we have predicted, hsa05215) and pancreatic cancer [53, 54].

Pathways of endometrial cancer (*hsa05213*), a malignant neoplasm involving the female genital system, have also been predicted to be associated with pancreatic cancer. The core regulatory factors of endometrial cancer and pancreatic cancer have quite a lot of crosstalk and overlap. Take *β*-catenin as an example, *β*-catenin has been revealed to participate in the Wnt signaling pathway in various tumor subtypes [55, 56]. The initiation and progression of both pancreatic cancer and endometrial cancer have been confirmed to be associated with Wnt signaling pathway, implying that such two regulatory networks may have crosstalk and our extracted KEGG pathway (hsa05213) may actually contribute to the progression of pancreatic cancer [57–59]. As for the other three important solid tumor associated pathways, two of them, renal cell carcinoma and colorectal cancer associated pathways (*hsa05211 *and* hsa05210*), contain functional regulatory genes and pathways that have been reported to contribute to pancreatic cancer at the same time. In renal cell carcinoma,* MET* has been reported to interact with the hepatocyte growth factor (HGF) and turns out be the initial signal for* MAPK* signaling pathway [60]. Coincidentally, in pancreatic cancer,* MET* has also been confirmed to be quite a crucial gene for tumor initiation, progression, and metastasis, implying the crosstalk of pathways associated pancreatic cancer and renal cell carcinoma [61, 62]. As for colorectal cancer associated pathways (hsa05211), during the tumorigenesis of colorectal cancer, chromosomal instability (CIN) has been revealed to be a core driver mechanism and pathogenesis of the initiation [63]. In pancreatic cancer, CIN has also been regarded as a common phenotype and pathogenic factor, implying the undergoing relationship between such two regulatory networks [64]. According to Table 1, we also obtained the specific KEGG pathway describing pancreatic cancer associated pathway (*hsa05212*) which is definitely associated with pancreatic cancer, validating the accuracy and efficacy of our prediction.

Apart from such solid tumor subtype, two leukemia subtypes and sarcoma associated pathways have also been obtained to contribute to the tumorigenesis of pancreatic cancer. KEGG pathway,* hsa05220*, describes pathogenic biological processes of chronic myeloid leukemia (CML). Various factors have been reported to contribute to the chronic myeloid leukemia. PI3K-AKT pathway has been identified as a core component of chronic myeloid leukemia associated pathways as we have predicted [65]. Based on recent publications, such pathway (PI3K-AKT pathway) has also been confirmed to be quite crucial for pancreatic cancer, validating our prediction [66]. Apart from that, the specific* BCR-ABL* fusion gene has also been identified in some pancreatic cancer patients, implying that* BCR-ABL* fusion gene may also contribute to the tumorigenesis of pancreatic cancer [67]. Apart from CML associated pathways, regulatory networks that contribute to another nonsolid tumor subtype, acute myeloid leukemia (AML) (*hsa05221*), have also been contained in our results. As we all know,* KRAS*,* STAT,* and their respective regulatory pathways have all been associated with our predicted pathway (hsa05221) [68, 69]. As we have mentioned above,* KRAS* has been identified as a core regulatory factor that contributes to pancreatic cancer [49]. According to recent publications,* STAT* as AML associated gene has also been reported to contribute to pancreatic cancer, validating our prediction of pancreatic cancer associated genes [70]. Glioma, rising from glial cells, is a malignant sarcoma involving the brain and central nervous system. Based on our results, glioma associated pathways (*hsa05214*) may also contribute to pancreatic cancer. Genes associated with glioma such as* EGFR* (as oncogene) and* PTEN* (as tumor suppressor) have also been reported to contribute to the initiation and progression of pancreatic cancer [71, 72].

*(2) Detailed Pathways That May Participate in Tumorigenesis*. Apart from pathways that directly describe the tumorigenesis, four KEGG pathways that describe the detailed pathways were also extracted. KEGG pathway,* hsa04150,* which describes the mTOR signaling pathway has been predicted to contribute to pancreatic cancer. The relationship between mTOR signaling pathway and pancreatic cancer has been revealed by multiple recent publications [73–75]. As a regulatory mechanism for cell proliferation, mTOR signaling pathway has been confirmed to have crosstalk with various core regulatory factors and their respective signaling pathways including* MAPK*,* TP53*, RAS, and* EGFR* [76–79]. Some of such regulatory factors have also been contained in our results.* MAPK* signaling pathway (*hsa04010*) has been confirmed to have crosstalk with mTOR signaling pathway as we have mentioned above and has been reported to be quite crucial for the invasion and metastasis of pancreatic cancer [80, 81]. Apart from such two functional signaling pathways, another pathway, which has been widely reported to contribute to endometrial cancer,* ERBB* signaling pathway (*hsa04012*), is also in Table 1 [82]. During the initiation and progression of pancreatic cancer,* ERBB* signaling pathway has been confirmed to participate in the biological processes, validating our newly presented algorithm [83]. Neurotrophins have firstly been identified as a group of proteins that contribute to the survival, development, and function of neurons [84]. However, recent publications have revealed that neurotrophins may participate in the survival and proliferation of various cell types including the tumor cells [85–87]. Such functional protein, neurotrophin, which is regulated by another functional gene* TRK* has also been reported to contribute to pancreatic cancer, validating the efficacy of our prediction [88]. In Table 1, a specific KEGG pathway,* hsa04722*, which describes the neurotrophin signaling pathway was also listed. Based on our analyses, such biological process may definitely contribute to pancreatic cancer.

*(3) Specific Pathways That Contribute to Cell-Cell Interaction*. The last three KEGG pathways with MI values no less than 0.01 have been confirmed to contribute to the cell-cell/cell-protein interaction associated pathways. KEGG pathway* hsa04510* describes the focal adhesion associated pathways. The abnormal activation of focal adhesion associated pathway has been widely reported in pancreatic cancer, implying that focal adhesion may be core biological processes during the tumorigenesis of pancreatic cancer [89]. Apart from focal adhesion, another biological process involving cell-cell interaction, T cell receptor signaling pathway (*hsa04660*), was also extracted in this study. It has been widely reported that the T cell receptor signaling pathway has been blocked or abnormally regulated in tumor microenvironment [90]. In pancreatic cancer, the initiation and progression of pancreatic cancer also interfere with the normal function of T cell receptor. Considering the recognition and cytolysis functions of T cells, the tumor cells and the T cells may put the selective pressure on each other and coevolve [91]. During the evolutionary processes, T cells with high recognition and cytolysis ability, which are both induced by T cell receptor signaling pathway, are all screened out, leaving dysfunctional T cells in tumor microenvironment [92, 93]. Such coevolution processes imply the regulatory role of T cell receptor signaling pathway in pancreatic cancer. We also obtained a functional signaling pathway (*hsa05160*) that is related with the infection of hepatitis C virus. Based on meta-analysis and case-control study, the infection of specific virus (hepatitis B and hepatitis C) has been confirmed to increase the risk of pancreatic cancer, validating the prediction based on our new algorithm, though the undergoing mechanism has not been fully revealed [94, 95].

#### 3.2.2. GO Terms Associated with Pancreatic Cancer

Apart from the KEGG pathways mentioned above, five GO terms (listed in Table 2) that describe different biological processes were also extracted in this study, which are also deemed to contribute to the tumorigenesis of pancreatic cancer. The detailed analyses are listed below.

GO:* 0048011*, which describes the* neurotrophin-TRK* receptor signaling pathway, has been predicted to contribute to pancreatic cancer. As we have mentioned above,* neurotrophin-TRK* receptor has been reported to contribute to the growth and progression of human pancreatic cancer [96]. Such evidence validates the efficacy and accuracy of our prediction algorithm. Apart from that, another GO term (GO:* 0016772*) describes the transferase activity, especially for the activity of transferring phosphorus-containing groups. During the progression of pancreatic cancer, transferases, especially for those that contribute to the transferring phosphorus-containing groups, have been identified to contain various variants and function abnormally. Take a classical transferase* SphK1* as an example,* SphK1* as a tumor associated protein has been reported to be overexpressed in pancreatic cancer [97]. Recent publications have confirmed that* SphK1* regulates the sphingolipid metabolism and further contributes to the resistance against gemcitabine, a widely used anticancer drug for pancreatic cancer, validating the underlying role of phosphorus associated transferase for pancreatic cancer [97]. Another GO term (GO:* 0016303*) describes the specific activity of 1-phosphatidylinositol-3-kinase (*PI3K*). As we have mentioned above,* PI3K* associated pathway has been widely reported to contribute to pancreatic cancer [66]. Similarly, the remaining two GO terms (GO:* 0004713* and GO:* 0007265*) describe the protein tyrosine kinase (*PTK*) activity and the* Ras* protein signal transduction, respectively, which have also been reported to contribute to pancreatic cancer. The inhibitors for protein tyrosine kinase have been widely used in clinical treatment for pancreatic cancer, implying the driving effect of* PTK* for pancreatic cancer [98]. As for Ras protein signal transduction, proteins of* Ras *family have been widely reported to contribute to tumorigenesis [3, 99]. A specific protein of* Ras* family,* K-RAS,* has been confirmed to be a driver gene for pancreatic cancer, validating the accuracy of our prediction.

According to the analyses listed above, all extracted functional KEGG pathways and GO terms are confirmed to definitely contribute to pancreatic cancer. Some new findings may give new insights for the study of pancreatic cancer or other types of cancer.

## 4. Conclusions

In this study, effective features, derived from the GO terms and KEGG pathways, were utilized to encode the genes related to pancreatic cancer. After being analyzed by the mRMR method, 22 key features were extracted, corresponding to five GO terms and 17 KEGG pathways. These GO terms and KEGG pathways may be the novel materials to investigate pancreatic cancer. Furthermore, they may also be useful to build an effective computational method for identification of novel genes related to pancreatic cancer. In future, we will try our best in this regard.

## Supplementary Material

Supplementary Material I lists 65 validated genes related to pancreatic cancer.
Supplementary Material II lists the MaxRel feature list obtained by mRMR method.

## Figures and Tables

**Table 1:** 17 important KEGG pathways for pancreatic cancer.

KEGG pathway ID	KEGG pathway	MI value	Rank in MaxRel feature list
hsa05211	Renal cell carcinoma	0.011	1
hsa04010	MAPK signaling pathway	0.011	3
hsa05212	Pancreatic cancer	0.011	4
hsa05200	Pathways in cancer	0.011	5
hsa05210	Colorectal cancer	0.011	6
hsa05214	Glioma	0.011	7
hsa05220	Chronic myeloid leukemia	0.011	8
hsa05223	Non-small-cell lung cancer	0.01	9
hsa04510	Focal adhesion	0.01	10
hsa05213	Endometrial cancer	0.01	11
hsa05221	Acute myeloid leukemia	0.01	12
hsa05215	Prostate cancer	0.01	13
hsa05160	Hepatitis C	0.01	14
hsa04012	ErbB signaling pathway	0.01	16
hsa04660	T cell receptor signaling pathway	0.01	18
hsa04150	mTOR signaling pathway	0.01	20
hsa04722	Neurotrophin signaling pathway	0.01	22

**Table 2:** Five important GO terms for pancreatic cancer.

GO term ID	GO term	MI value	Rank in MaxRel feature list
GO: 0007265	Ras protein signal transduction	0.011	2
GO: 0048011	Neurotrophin-TRK receptor signaling pathway	0.01	15
GO: 0016772	Transferase activity, transferring phosphorus-containing groups	0.01	17
GO: 0016303	1-Phosphatidylinositol-3-kinase activity	0.01	19
GO: 0004713	Protein tyrosine kinase activity	0.01	21
