# Supplementary material for: Analysis of Important Gene Ontology Terms and Biological Pathways Related to Pancreatic Cancer
Source: Biomed Res Int. 2016 Nov 9;2016:7861274. doi: 10.1155/2016/7861274 (PMC5120232; doi:10.1155/2016/7861274)
Supplement: Supplementary file 1 — Supplementary Material I lists 65 validated genes related to pancreatic cancer. Supplementary Material II lists the MaxRel feature list obtained by mRMR method. [file 7861274.f1.zip › Supp_I.docx]

**Supplementary Material I**. 65 validated genes related to pancreatic cancer.

| **Index** | **Gene name** | **Ensembl ID** |
| --- | --- | --- |
| 1 | SMAD2 | ENSP00000262160 |
| 2 | PLD1 | ENSP00000342793 |
| 3 | TGFA | ENSP00000295400 |
| 4 | RELA | ENSP00000384273 |
| 5 | PIK3R3 | ENSP00000262741 |
| 6 | CDC42 | ENSP00000314458 |
| 7 | AKT2 | ENSP00000375892 |
| 8 | TGFB1 | ENSP00000221930 |
| 9 | SMAD4 | ENSP00000341551 |
| 10 | E2F3 | ENSP00000262904 |
| 11 | RAF1 | ENSP00000251849 |
| 12 | BRAF | ENSP00000288602 |
| 13 | BAD | ENSP00000309103 |
| 14 | BRCA2 | ENSP00000369497 |
| 15 | MAPK8 | ENSP00000353483 |
| 16 | E2F2 | ENSP00000355249 |
| 17 | RALA | ENSP00000005257 |
| 18 | MAP2K1 | ENSP00000302486 |
| 19 | RALB | ENSP00000272519 |
| 20 | ERBB2 | ENSP00000269571 |
| 21 | EGF | ENSP00000265171 |
| 22 | CDKN2A | ENSP00000355153 |
| 23 | PIK3CA | ENSP00000263967 |
| 24 | SMAD3 | ENSP00000332973 |
| 25 | MAPK3 | ENSP00000263025 |
| 26 | VEGFA | ENSP00000361125 |
| 27 | IKBKB | ENSP00000339151 |
| 28 | PIK3CB | ENSP00000289153 |
| 29 | RB1 | ENSP00000267163 |
| 30 | PIK3R5 | ENSP00000269300 |
| 31 | TGFB2 | ENSP00000355896 |
| 32 | IKBKG | ENSP00000358622 |
| 33 | MAPK1 | ENSP00000215832 |
| 34 | RALGDS | ENSP00000361120 |
| 35 | STAT1 | ENSP00000354394 |
| 36 | RAC2 | ENSP00000249071 |
| 37 | CCND1 | ENSP00000227507 |
| 38 | ARAF | ENSP00000366244 |
| 39 | JAK1 | ENSP00000343204 |
| 40 | RAC1 | ENSP00000348461 |
| 41 | RAD51 | ENSP00000267868 |
| 42 | RAC3 | ENSP00000304283 |
| 43 | RALBP1 | ENSP00000019317 |
| 44 | BCL2L1 | ENSP00000302564 |
| 45 | CDK4 | ENSP00000257904 |
| 46 | MAPK10 | ENSP00000352157 |
| 47 | AKT3 | ENSP00000263826 |
| 48 | E2F1 | ENSP00000345571 |
| 49 | ARHGEF6 | ENSP00000250617 |
| 50 | PIK3CD | ENSP00000366563 |
| 51 | TGFBR2 | ENSP00000351905 |
| 52 | TGFBR1 | ENSP00000364133 |
| 53 | CDK6 | ENSP00000265734 |
| 54 | EGFR | ENSP00000275493 |
| 55 | AKT1 | ENSP00000270202 |
| 56 | CASP9 | ENSP00000330237 |
| 57 | STAT3 | ENSP00000264657 |
| 58 | MAPK9 | ENSP00000321410 |
| 59 | NFKB1 | ENSP00000226574 |
| 60 | CHUK | ENSP00000359424 |
| 61 | PIK3R2 | ENSP00000222254 |
| 62 | PIK3CG | ENSP00000352121 |
| 63 | TP53 | ENSP00000269305 |
| 64 | TGFB3 | ENSP00000238682 |
| 65 | PIK3R1 | ENSP00000274335 |
